# The role of accent imitation in sensorimotor integration during processing of intelligible speech

**DOI:** 10.3389/fnhum.2013.00634

**Published:** 2013-10-04

**Authors:** Patti Adank, Shirley-Ann Rueschemeyer, Harold Bekkering

**Affiliations:** ^1^Department of Speech, Hearing and Phonetic Sciences, Division of Psychology and Language Sciences, University College LondonLondon, UK; ^2^Donders Institute for Brain, Cognition and Behaviour, Radboud University NijmegenNijmegen, Netherlands; ^3^Department of Psychology, University of YorkYork, UK

**Keywords:** imitation, fMRI, speech, accent, sensorimotor

## Abstract

Recent theories on how listeners maintain perceptual invariance despite variation in the speech signal allocate a prominent role to imitation mechanisms. Notably, these simulation accounts propose that motor mechanisms support perception of ambiguous or noisy signals. Indeed, imitation of ambiguous signals, e.g., accented speech, has been found to aid effective speech comprehension. Here, we explored the possibility that imitation in speech benefits perception by increasing activation in speech perception and production areas. Participants rated the intelligibility of sentences spoken in an unfamiliar accent of Dutch in a functional Magnetic Resonance Imaging experiment. Next, participants in one group repeated the sentences in their own accent, while a second group vocally imitated the accent. Finally, both groups rated the intelligibility of accented sentences in a post-test. The neuroimaging results showed an interaction between type of training and pre- and post-test sessions in left Inferior Frontal Gyrus, Supplementary Motor Area, and left Superior Temporal Sulcus. Although alternative explanations such as task engagement and fatigue need to be considered as well, the results suggest that imitation may aid effective speech comprehension by supporting sensorimotor integration.

## Introduction

In everyday communication, humans have to deal with a variety of challenging listening situations, such as the presence of background noise, poor telephone connections, or unfamiliar accents. Recent studies have investigated cognitive and neural mechanisms underlying the ability to process speech effectively in such challenging listening situations (Davis and Johnsrude, 2003; Rodd et al., 2005, 2010; Obleser et al., 2007, 2011; Adank and Devlin, 2010; Obleser and Kotz, 2010; Peelle et al., 2010; Adank, 2012). The aforementioned studies have established that effective speech comprehension recruits areas involved in speech perception including left Superior Temporal Sulcus (STS), areas involved in linguistic and articulatory processes including left Inferior Frontal Gyrus (IFG), areas involved in speech production including Precentral Gyrus and Supplementary Motor Area (SMA).

There is growing consensus that cortical regions associated with speech production, such as ventral premotor cortex, IFG, SMA/pre-SMA, and primary motor cortex play an active and essential role in effective speech comprehension (Skipper et al., 2006; D'Ausilio et al., 2009; Londei et al., 2009; Callan et al., 2010; Tremblay and Small, 2011; Adank, 2012). Despite this emerging agreement (but see Venezia et al., 2012), much is still unclear about precisely how speech motor and premotor regions contribute to the comprehension process.

Recent theories suggests that mental simulation of perceived actions may aid listeners when predicting upcoming speech signals (Wilson and Knoblich, 2005; Pickering and Garrod, 2007). Simulation theories of action perception argue that observing actions results in the automatic generation of motor plans required to perform these actions. The motor plans are used to produce a forward model of the incoming action. These forward models use information about the movement properties of muscles to generate a simulated course of movement in parallel with, or even in anticipation of, the perceived movement (Grush, 2004). Any discrepancy between the simulated movement from the forward model and the real-world movement results in prediction errors and leads to corrective commands. This type of forward model serves to anticipate others' (speech) actions as if they were produced by the observer (Locatelli et al., 2012). Forward models thus, generate a series of predictions that may be used as disambiguating information in situations of when action perception is made more difficult due to noisy or ambiguous observing conditions (Wilson and Knoblich, 2005; Pickering and Garrod, 2007). For speech, these type of conditions involve listening to speech in the presence of background noise, or listening to someone speak with an unfamiliar regional accent (Adank et al., 2009).

Some propose that the prediction signal generated by the forward models is optimized for the type of actions the observers have had experience executing themselves (Knoblich and Sebanz, 2006; Grafton, 2009). Behavioral evidence for this prediction comes from a study on basketball players by Aglioti et al. (2008). Aglioti et al. compared elite basketball players (experts at observing the action and also at performing the action) with a control group (basketball referees; experts at observing the action but not playing themselves) on how effectively each group could judge whether a basketball would be thrown in the basket. They showed that being experienced in performing an action such as throwing a basketball allows one to efficiently predict the outcome of other players throwing a basketball.

For speech, we recently found that short-term experience with speaking in an unfamiliar regional accent helps comprehension of sentences spoken in that accent (Adank et al., 2010). Adank et al.'s participants listened to sentences spoken in an unfamiliar accent in background noise in a pre-test phase and verbally repeated key words. Next, participants were divided into six groups and either received no training, listened to sentences in the unfamiliar accent without speaking, repeated the accented sentences in their own accent, listened to and transcribed the accented sentences, listened to and imitated the accented sentences, or listened to and imitated the accented sentences without being able to hear their own vocalizations. Post-training measures showed that participants who imitated the speaker's accent could repeat key words in poorer signal-to-noise ratios (i.e., under more challenging listening conditions) than participants who had not imitated the accent. Adank et al.'s results demonstrate that having experience with speaking in a specific way (i.e., in an unfamiliar accent) can positively affect speech processing by optimizing comprehension of sentences spoken in a similar way.

The neural underpinnings of the optimizing effect of experience with performing an action on action perception have been investigated a several fMRI experiments (Calvo-Merino et al., 2005; Wilson and Iacoboni, 2006; Lahav et al., 2007). Calvo-Merino et al. (2005) studied professional dancers and compared neural activity when male and female dancers viewed dance moves that were performed by their own gender (i.e., moves they would perform when dancing themselves), compared to other moves performed by the other gender (i.e., moves they would always observe, but not perform, when dancing themselves). Calvo-Merino's design allowed them to separate brain responses related to dance motor representation from those related to visual knowledge about the observed moves. Left dorsal premotor cortex, left inferior parietal sulcus, and right cerebellum showed a higher Blood Oxygenated-Level Dependent (BOLD-) response for dance moves they would perform themselves than for observed moves.

Lahav et al. (2007) trained non-musicians in playing several simple melodies on the piano. Participants were scanned while listening to pieces they had learnt to play, plus pieces they had not learnt. An increased BOLD-response was found in bilateral IFG, the posterior middle premotor region and the inferior parietal lobule (IPL) (Supramarginal gyrus and Angular gyrus) bilaterally and left cerebellum.

Finally, Wilson and Iacoboni tested neural responses for 5 native and 25 non-native speech sounds varying in how easy they were to produce for their native American-English speaking participants. They report increased activation in bilateral superior temporal areas for foreign speech sounds that are more difficult to produce. No correlations were found between the ease with which the foreign sounds could be produced in speech motor areas. Nevertheless, in a region-of-interest analysis, they report that the BOLD-response in premotor cortex, a speech motor area, was elevated for listening to foreign speech sounds compared to native speech sounds. The results from Wilson and Iacoboni thus, contradict the expectation that having had more experience with a motor action (which is the case for native speech sounds) activates motor areas to a higher degree. However, Wilson did not explicitly evaluate how experience with producing the foreign speech sounds affected brain activation when listening to those speech sounds. Thus, it remains unclear how speech motor experience affects neural activation during speech comprehension.

The present study will examine how motor experience with imitating a novel accent affects the activation of cortical areas associated with speech perception and production when subsequently listening to the accented speech in background noise. Based on Adank et al.'s behavioral study, we hypothesized that speech imitation experience supports speech comprehension through the integrating of information from specific speech perception and production areas.

We mapped out the neural bases associated with increased robustness of speech comprehension after imitating an unfamiliar accent and after repeating the accented speech in one's own accent. Two groups of listeners were scanned using an adapted version of the staircase procedure described in Adank et al. (2010). In a pre-test, participants were examined on their comprehension of sentences spoken in an unfamiliar accent in background noise. Next, one group of participants repeated a series of accented sentences in their own accent, while the second group vocally imitated the sentences in the unfamiliar accent in a training session. Finally, both groups were tested again on their comprehension of accented speech in background noise. If imitation supports sensorimotor integration, we expected a different pattern of activation of cortical areas associated with either speech comprehension or speech production for listeners who have had experience with imitating the unfamiliar accent during the training session. We focused on three left-lateralized regions, namely posterior STS, IFG, and SMA, all of which have been associated with speech perception and speech production tasks (for STS: Scott et al., 2000; Blank et al., 2002; Narain et al., 2003; Crinion and Price, 2005; Tremblay and Gracco, 2009, for IFG: Davis and Johnsrude, 2003; Tremblay and Gracco, 2009; Adank and Devlin, 2010, for SMA: Alario et al., 2006; Wong et al., 2008; Fridriksson et al., 2009).

## Materials and methods

### Participants

We tested 36 participants (23F and 13M, mean 21.8 years, *SD* 2.9 years, range 18–29 years). All participants were right-handed, native speakers of Dutch, with no history of oral or written language impairment, or neurological or psychiatric disease. All gave written informed consent and were paid for their participation. The study was approved by the local ethics committee of the Radboud University Nijmegen. Four participants were excluded from further analysis: one participant (F) originally allocated to the imitation group was excluded as the second run was not collected due to technical difficulties, another participant allocated to the imitation group (M) was excluded due to an abnormality in his structural scan, a repeat group participant (M) was excluded as his scan was interrupted due to him feeling unwell, and a final participant (F) in the repeat group was excluded due to poor performance on the behavioral task; her results showed more than 50% missed responses. All analyses were run on the results from the remaining 32 participants. The repeat group consisted of 15 participants (9F and 6M, mean 21.6 years, *SD* 3.2 years, range 18–29 years) and the imitation group consisted of 17 participants (10F and 6M, mean 22.2 years, *SD* 2.8 years, range 18–28 years).

### Stimulus material

The test stimulus set consisted of 96 sentences (see Table A1 in the Appendix) that were taken from the speech reception threshold (SRT) corpus (Plomp and Mimpen, 1979). This corpus has been widely used for assessing intelligibility of different types of stimuli, for example, for foreign-accented speech (Van Wijngaarden et al., 2002). Sentences in the SRT corpus are designed to resemble short samples of conversational speech. All consist of maximally eight or nine syllables and do not include words longer than three syllables. The 48 sentences used in the training (listed in Table A2) were taken from an earlier study on comprehension of accented speech (Adank et al., 2012) and produced by the same speaker as the 96 test sentences. Finally, 30 sentences were recorded in Standard Dutch and used in a hearing pre-test (cf. Table A3).

All sentences were spoken by a single female speaker of Standard Dutch. The test sentences were recorded in both Standard Dutch and an unfamiliar, novel accent. The training sentences were recorded in the novel accent only. The novel accent was created by instructing the speaker to read sentences with an adapted orthography (see also Adank et al., 2010). This accent was designed to sound different from Standard Dutch and not intended to replicate an existing Dutch accent. The orthography was systematically altered to achieve changes in all 15 Dutch vowels as listed in Table 1. Only vowels bearing primary or secondary stress were included in the orthography conversion. An example of a sentence in Standard Dutch and a converted version is given below, including a broad phonetic transcription using the International Phonetic Alphabet (IPA, 1999):

**Table 1:**
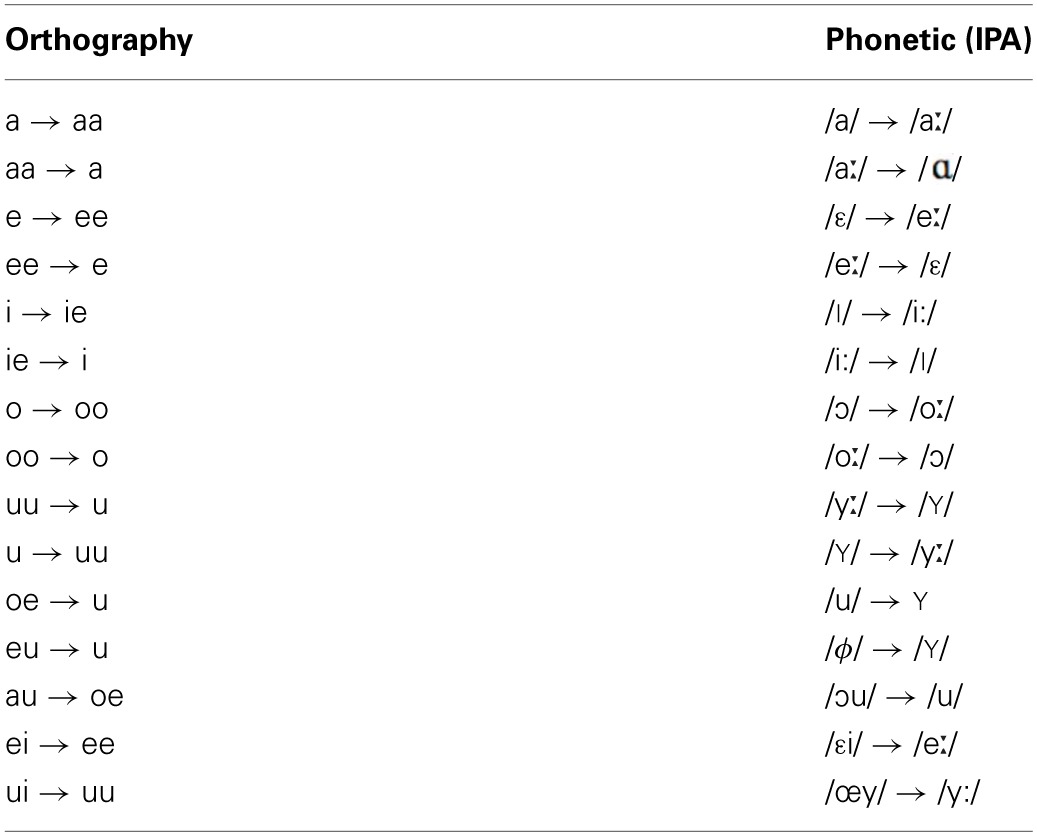
**Intended vowel conversions for obtaining the novel accent**.

The left column shows the altered orthography in Standard Dutch, and the right column shows the intended change in pronunciation of the vowel in broad phonetic transcription, using the International Phonetic Alphabet (IPA, 1999).

Standard Dutch: “De bal vloog over de schutting”
                          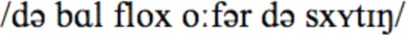                          [The ball flew over the fence]
After conversion: “De baal flog offer de schuuttieng”
                          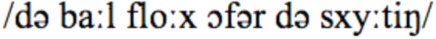

The stimulus materials used in the scanner was created as follows. The sentences in background noise were created by adding continuous speech-shaped noise to the accented sentences in quiet at so that the final signal-to-noise ratio (SNR) was set to −2 dB, 0 dB, +2 dB, +4 dB, +6 dB, +8 dB, +10 dB, +12 dB, +14 dB, or +16 dB, thus, resulting in 10 versions for each sentence. Speech-shaped noise was added using Matlab (Mathworks Inc.). An acoustically matched—yet unintelligible—baseline version of each sentence was created by spectrally rotating (Blesser, 1972) and time-reversing the sentence, using Praat (Boersma and Weenink, 2003). No noise was added to the baseline sentences. Sentences in all conditions were subsequently saved at 70 dB SPL.

### MRI data acquisition

Whole-brain imaging was performed at the Donders Centre for Brain, Cognition and Behavior, Centre for Cognitive Neuroimaging, at a 3T MR scanner (Magnetom Trio, Siemens Medical Systems, Erlangen, Germany), in Nijmegen, The Netherlands. The sentences were presented over sound-attenuating (~30 dB SPL) electrostatic headphones (MRConFon, Magdeburg, Germany) during continuous scanner acquisition (GE-EPI, echo time 35 ms; 32 axial slices; voxel size 3.5 × 3.5 × 3.5 mm, slice thickness 3 mm, inter-slice gap 0.5 mm; field of view 224 mm; flip angle 70). All functional images were acquired in two runs. Functional scans were obtained every 2 s in a continuous scanning paradigm (TR 2 s). Listeners watched a fixation cross that was presented on a screen and viewed through a mirror attached to the head coil. After the acquisition of functional images, a high-resolution structural scan was acquired (T1-weighted MP-RAGE, 192 slices, echo time 3.93 ms; field of view 256 mm, slice thickness 1 mm). Finally, a diffusion-weighted scan was acquired with 68 directions (not included in the present analysis). Total scanning time was around 60 min.

### Procedure

**Figure 1:**
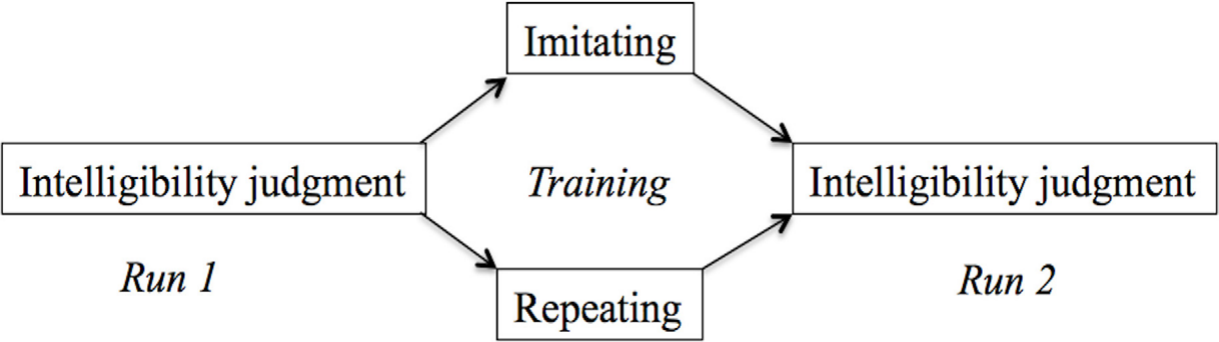
**Tasks in the three phases of the experiment**. Both groups performed an intelligibility judgment in the first run. Subsequently, the imitation group imitated a series of sentences in an unfamiliar accent, while the repeat group repeated sentences in their own accent. Finally, all participants performed the intelligibility judgment task again.

The experiment had a mixed-subject design with two groups (Figure 1): Repeat or Imitate. Participants were randomly allocated to the Repeat or Imitation group. Imaging data was collected in two runs and participants performed a training task in between these runs. Before the start of the scanning session, participants completed a hearing test in a quiet room. This test established the SRT as described in Plomp and Mimpen (1979) for sentences spoken in Standard Dutch by the same female speakers as used for the test in the scanner. Using a similar procedure as in (Adank et al., 2010), participants responded by repeating back what they had heard and the experimenter scored key words (see Table A1 in the Appendix). In this “standard” version, of the SRT, participants were presented the first sentence at +10 dB SNR. If they correctly repeated more than two out of four keywords, the next sentence was played at +2 dB SNR, if they got more than two key words in this sentence correct, the next sentence was played at −6 dB. This procedure continued until the participant either got two keywords right, or if he or she got fewer than two keywords right. If they repeated two keywords correctly, the next sentence was played at the same SNR as the previous sentence, while the SNR was increased with +2 dB if fewer than two keywords were repeated. If the participant than got more than two keywords right, the SNR decreased in steps of −2 dB. This staircase procedure was repeated for 30 sentences (Table A3). The final SNR, or SRT, was calculated as the average across all instances for which a reversal occurred, following Adank et al. (2010) and Plomp and Mimpen (1979). A reversal occurs whenever the SNR changed direction (e.g., from 0 dB SNR to +2 db SNR, or to −2 dB SNR). The participants showed an average SRT of −3.69 dB (0.95 dB *SD*), indicating that their hearing was in the normal range for participants with normal hearing (Plomp and Mimpen, 1979).

While in the scanner, participants in both groups performed an intelligibility judgment in the first run. Following the procedure in Adank et al. (2010), participants in the repeat group were instructed to listen to each accented sentence and then to repeat it in their own accent, without imitating the accent. Participants were explicitly instructed not to imitate the speaker's accent. If participants imitated the accent, they were reminded by the experimenter to repeat the sentence in Standard Dutch. Participants repeated 48 sentences. In the imitation group, participants were instructed to imitate vocally the precise pronunciation of the accented sentence. If participants repeated the sentence in their own accent, the experimenter instructed to imitate the accent as they heard it spoken. Participants imitated 48 sentences during the training and training was not attested using a formal scoring device. The scanner was turned off during the training phase. Next, the second run commenced and participants performed the intelligibility judgment task from run 1 for another series of accented sentenced. A total of 96 test sentences was presented across the two runs, plus 48 training sentences in between runs. Per run, 24 acoustically-matched baseline stimuli and 48 test sentences were presented. The distribution of the test sentences and baseline stimuli (which were created based on the 96 test sentences) were counterbalanced across runs and participants.

A single trial in the intelligibility judgment task began with a tone signal of 250 Hz with a duration of 200 ms, followed by a pause of 200 ms, followed by the presentation of a sentence. After the sentence was presented, participants judged its intelligibility, using a button-box with four buttons (one per finger) that they were holding in their right hand. If they found the sentence completely unintelligible, they pressed with their index finger (score 1), if they found the sentence slightly more intelligible they pressed the button under their middle finger (score 2), if they found the sentence intelligible they pressed with their ring finger (score 3) and if they found the sentence very intelligible they pressed the button under their little finger (score 4). This procedure was the same across participants. The SNR of the following sentence depended on the score of the previous sentence. If the participant had rated a specific sentence as 1 or 2, the next sentence was played at an easier (higher) SNR, and if the participant had rated a specific sentence as 3 or 4, the next sentence was played at a less favorable SNR. For instance, if a participant heard a sentence with a SNR of +4 dB and rated the sentence as unintelligible (score 2), then SNR for the next sentence was increased with 2 dB and the next sentence was presented at +6 dB. Alternatively, if a participant heard a sentence with a SNR of +4 dB and rated the sentence as very intelligible (score 4), then SNR for the next sentence was decreased with 2 dB and the next sentence was presented at +2 dB. Each run started with a sentence presented at +10 dB. The SNR decreased in steps of 2 dB until reaching −2 dB SNR and increased in steps of 2 dB up to +16 dB SNR. If these limits were reached, the SNR stayed the same until the participant pressed 3 or 4 (for +16 dB) or 1 or 2 (for −2 dB). As in the behavioral test outside the scanner, SRT was calculated as the average across all instances for which a reversal occurred. This procedure was identical across both runs. Intensity of stimulus presentation was set at a comfortable level for each participant in a familiarization session in which six sentences in Standard Dutch in quiet spoken by the same speaker (not included in the both runs experiment) were presented while the scanner was running. Stimulus presentation was performed using Presentation (Neurobehavioral Systems, Albany, CA), running on a Pentium 4 with 2 GB RAM, and a 2.8 GHz processor. The two runs plus training lasted ~40 min.

### Data processing

The neuroimaging data were pre-processed and analyzed using SPM8 (Wellcome Imaging Department, University College London, London, UK). The first four volumes of every functional run from each participant were excluded from the analysis to minimize T1-saturation effects. Next, the image time series were spatially realigned using a least-squares approach that estimates six rigid-body transformation parameters (Friston et al., 1995) by minimizing head movements between each image and the reference image, that is, the first image in the time series. Next, the time series for each voxel was temporally realigned to acquisition of the middle slice. Subsequently, images were normalized onto a custom Montreal Neurological Institute (MNI)-aligned EPI template (standard in SPM8) using both linear and non-linear transformations and resampled at an isotropic voxel size of 2 mm. All participants' functional images were smoothed using an 8 mm FWHM Gaussian filter. Each participants' functional image was processed using a unified segmentation procedure as implemented in SPM8. After segmentation of the T1 structural image (using the unified segmentation model) and co-registration to the mean functional image (Ashburner and Friston, 1997), functional images were spatially normalized into standard stereotaxic space of the MNI using segmentation parameters of the T1 structural image. A high-pass filter was applied with a 0.0078 Hz (128 s) cut-off to remove low-frequency components from the data, such as scanner drifts. The fMRI time series were analyzed within the context of the General Linear Model using an event-related approach, including an autoregressive AR (1) model during Classical (ReML) parameter estimation.

Three events of interest were identified and entered into a subject-specific General Linear Model: acoustically matched baseline sentences (Baseline), Unintelligible Speech (i.e., all sentences rated 1 or 2 by the participant), Intelligible Speech (i.e., all sentences rated 3 or 4 by the participant) for each run. Parameter estimates were calculated for each voxel, and contrast maps were constructed for each participant. The statistical model also considered six separate covariates describing the head-related movements (as estimated by the spatial realignment procedure). Linear-weighted contrasts were used to specify the main contrasts.

Intelligibility (having two levels: Intelligible Speech and Baseline), Run (Run 1 or Run 2), and Group (Repeat and Imitate) were analyzed in a 2 × 2 × 2 mixed ANOVA design, with Run as a within-subject and Group as a between-subject factor. We first identified areas showing a higher BOLD response for Intelligible Speech than for Baseline. Second, we identified areas that showed a higher BOLD-response as a result of having imitated during the training. We compared the activation across both runs for both groups. We used the following contrast to identify areas that showed a higher BOLD-response for the Imitate group than the Repeat group by directly comparing the second to the first run for each group: [Intelligible speech Imitation Group Run 2 > Intelligible speech Imitation Group Run 1] > [Intelligible speech Repeat Group Run 2 > Intelligible speech Repeat Group Run 1] (in short: [Imitation Run 2 > 1] > [Repeat Run 2 > 1]). Second, we identified areas showing a higher BOLD-response as a result of having repeated vs. imitated during the training using the contrast: [Intelligible speech Repeat Group Run 2 > Intelligible speech Repeat Group Run 1] > [Intelligible speech Imitation Group Run 2 > Intelligible speech Imitation Group Run 1] (in short: [Repeat Run 2 > 1]> [Imitation Run 2 > 1]). We also tested the contrasts [Imitate Run 2 > Run 1] and [Repeat Run 2 > Run 1] to get a general idea of areas that showed increases in BOLD-response across runs for both groups.

Significant activations in the whole-brain analysis were assessed with a Family Wise Error (FWE) correction for multiple comparisons (*p* < 0.05), in combination with a minimal cluster extent of 20 voxels. Finally, anatomical localization was performed using the Anatomy toolbox in SPM8 (Eickhoff et al., 2005) and verified with MRICRON using the ch2 template in MNI space (Rorden and Brett, 2000).

## Results

### Behavioral results

**Figure 2:**
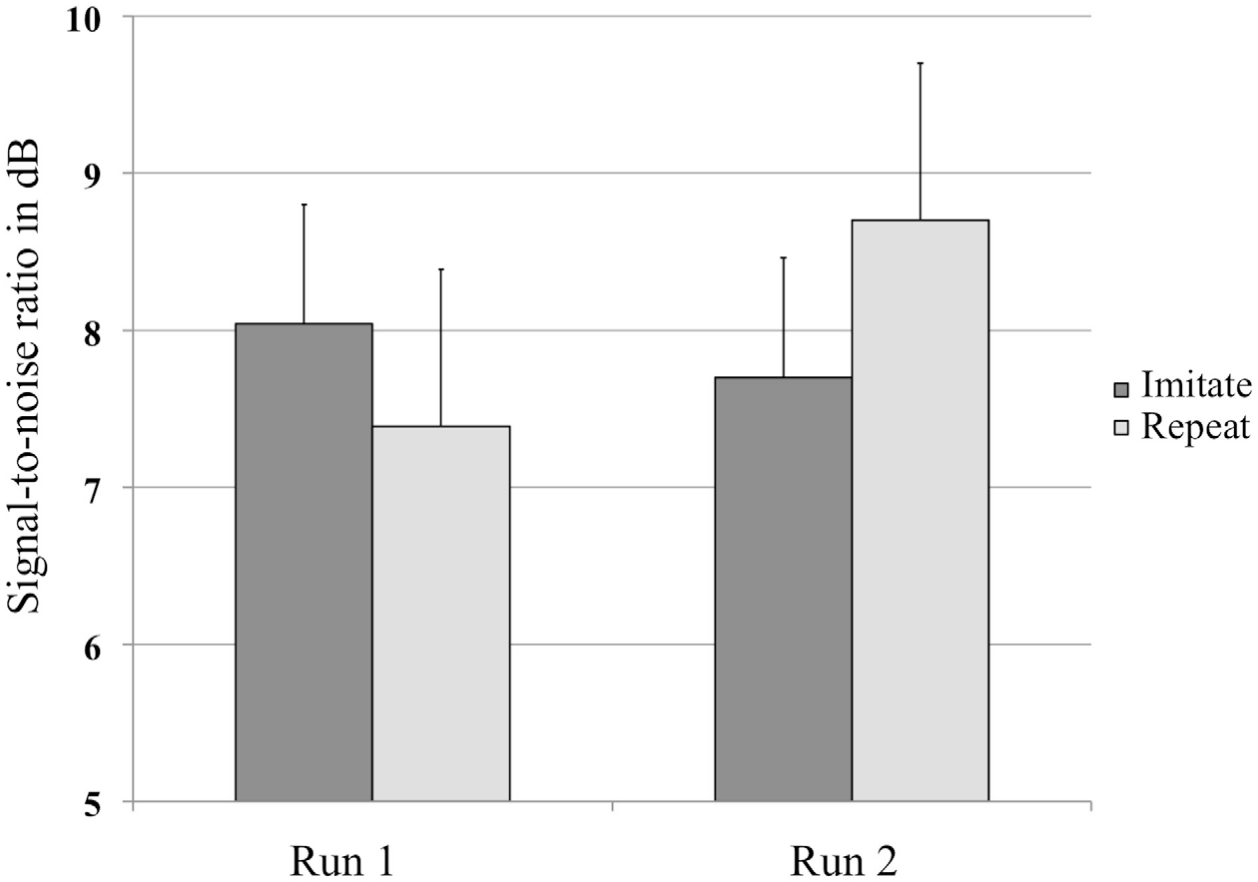
**Average values in decibel (dB) at which the sentences were presented in the experiment for both groups, before and after training**. Error bars represent one standard error.

A repeated-measures ANOVA with Group (Repeat or Imitate) and Run (1 or 2) on the SRT value in dB per run (Figure 2) revealed no main effects, but did show an interaction between Run and Group [*F*_(1, 30)_ = 6.21, *p* = 0.018, η^2^_*p*_ = 0.17]. The absence of a main effect of Run indicated that both groups of listeners judged the sentence materials to be equally intelligible when played at similar SNRs. The presence of an interaction between Run and Group indicates that listeners in both groups were affected differently by the training. Two *post-hoc* analyses showed a marginally significant trend for the repeat group to require more favorable SNRs in the second run [*t*_(14)_ = −2.033, *p* = 0.061], while no such trend was found for the imitate group [*t*_(16)_ = 0.946, *p* = 0.358].

### Neuroimaging results

Table 2 and Figure 2 show the network of areas involved in processing intelligible speech (the contrast Intelligibility included all stimuli that had been rated as 3 or 4 in the intelligibility judgment test) vs. the acoustically matched baseline. Intelligible speech was associated with higher BOLD values with peak coordinates in left and right anterior and posterior STS, left IFG, left insula, and SMA.

**Table 2:** **Activation for peaks separated by at least 8 mm for the whole-brain analysis for the contrasts [Intelligibility > Baseline], [Imitation Run 2 > 1] > [Repeat Run 2 > 1], and [Run 1] > [Run 2]**.

**Location**	**mm^3^**	***t***	**Equivalent *Z***	**MNI**
**[INTELLIGIBILITY > BASELINE]**				
Left STS	966	10.03	Inf^*^	−62 −32 4
Left STG		6.46	5.97	−58 −4 −8
Left MTG		5.31	5.02	−60 −16 −8
Left IFG (Triangularis)	306	6.70	6.17	−50 18 24
Left IFG (Orbitalis)	374	6.61	6.09	−46 28 −4
Left Insula		6.00	5.60	−36 22 −4
Right STG	250	5.94	5.55	56 −20 −6
Right STG		5.83	5.46	60 2 −12
Left SMA	71	5.32	5.03	−2 10 60
**[IMITATION RUN 2 > 1] > [REPEAT RUN 2 > 1]**				
Left STS	750	8.47	7.49	−62 −32 2
Left MTG		6.89	6.31	−58 −20 −8
Left STS		5.44	5.13	−58 −8 −8
Left IFG (Triangularis)	164	6.42	5.94	−52 18 26
Left SMA	20	5.30	5.01	−4 10 64
Left IFG (Orbitalis)	26	5.28	4.99	−46 26 −4
**[IMITATE RUN 2 > RUN 1]**				
Left MTG	6131	15.79	Inf	−58 −34 2
Left STG		14.18	Inf	−58 −2 −8
Left anterior insula		12.66	Inf	−30 24 −4
Left SMA	1448	11.90	Inf	−4 20 46
R ACC		6.63	6.11	12 30 28
L ACC		5.67	5.32	−8 32 22
Right STG	1512	11.42	Inf	60 2 −10
Left anterior insula		10.89	Inf	32 24 −2
Right TP		9.06	Inf	52 12 −18
White matter	680	10.49	Inf	−14 6 6
White matter	375	9.33	Inf	12 10 0
Right STG	47	6.11	5.69	46 −36 2
White matter	45	5.62	5.28	−4 −24 −18
White matter		4.80	4.58	−6 −20 −10
Left FFG	23	5.51	5.20	−38 −38 −20

Coordinates in MNI standard space. ACC, Anterior Cingulate Cortex; FFG, Fusiform Gyrus; IFG, Inferior Frontal Gyrus; MTG, Middle Temporal Gyrus; SMA, Supplementary Motor Area; STG, Superior Temporal Gyrus; STS, Superior Temporal Gyrus; TP, Temporal Pole.

* Inf denotes Equivalent Z > 10.0.

**Figure 3:**
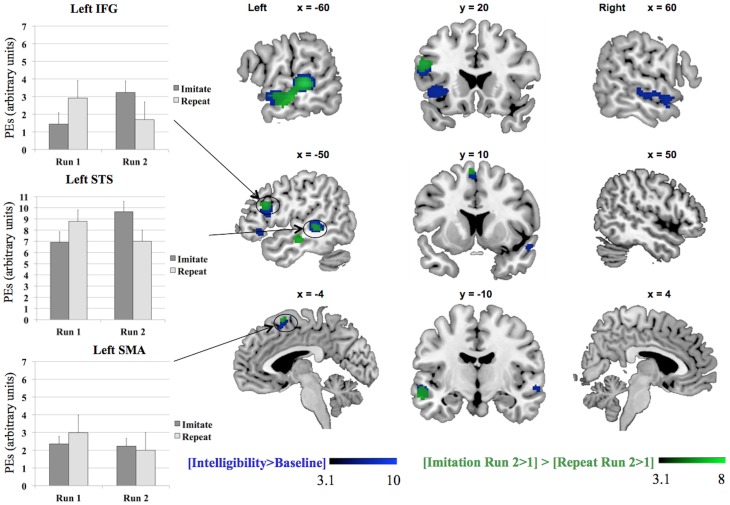
**Results for the whole-brain analysis for the contrasts [Intelligibility > Baseline] and [Imitation Run 2 > 1] > [Repeat Run 2 > 1], including plots of the parameter estimates (PEs) per group, IFG = Inferior Frontal Gyrus, SMA = Supplementary Motor Area, STS = Superior Temporal Sulcus**. The MarsBaR toolbox (Brett et al., 2002) was used to construct spheres, each with a radius of 10 mm, Left IFG (−50 18 26), left SMA (−4 10 64), and left STS (−62 −32 2). The parameter estimates in the three charts represent summary time courses per sphere. The legends represent *Z*-scores.

We tested the contrasts [Imitation Run 2 > 1] > [Repeat Run 2 > 1] and [Repeat Run 2 > 1] > [Imitation Run 2 > 1] to probe for areas showing an effect of training in the second run for both groups (see Table 2 and Figure 3) in a whole-brain analysis. The areas showing an increased BOLD-response for the contrast [Imitation Run 2 > 1] > [Repeat Run 2 > 1] effect included left STS, an area in left IFG, and a cluster in left SMA. For the reverse contrast [Repeat Run 2 > 1] > [Imitation Run 2 > 1] no significant clusters were found. However, at a less stringent significance level of *p* < 0.001, we found two clusters in left and right Supramarginal Gyrus (SMG) at [−62 −38 36] (157 voxels) and [60 −42 22] (50 voxels). Finally we found no supra-threshold voxels for the contrast [Repeat Run 2 > Run 1], while for [Imitate Run 2 > Run 1] significant clusters were found in MTG bilaterally, left STG, SMA, bilateral Anterior Insula, Right Temporal Pole, and left Fusiform Gyrus.

## Discussion

### Main findings

We investigated the neural bases associated with increased robustness of speech comprehension after imitating an unfamiliar accent and after repeating the accented speech in one's own accent. The aim of the study was to establish the effect of short term-experience with imitating accented speech on the activation of cortical areas associated with speech perception and production when subsequently listening to the accent in background noise.

Based on previous studies on the role of motor experience, it was expected that motor experience with speaking in a novel unfamiliar accent action would result in a changed activation pattern brain areas associated with speaking and listening to speech, including left IFG, STS, and SMA. Previous studies on motor experience in general action processing (Calvo-Merino et al., 2005; Lahav et al., 2007) predicted that experience with performing a motor act (such as performing a dance move in Calvo-Merino et al.) increased activation in motor areas associated with performing that act while participants passively observed the act. However, a previous study on the effect of long-term speech production experience on speech sound perception (Wilson and Iacoboni, 2006) found that activation in premotor cortex was elevated for listening to foreign speech sounds.

Our results show that motor experience with accented speech leads to increased activation in speech perception and speech motor areas during subsequent comprehension of that accent. Areas in left STS, left IFG, and left SMA showed an interaction between run and group: they were more active during the second run for the imitation group compared to the difference between the first and second run for the repeat group. This effect was supported by a significant behavioral interaction between Run and Group.

Interestingly, these results are largely in line with previous studies that established the effect of perceptual experience with unfamiliar or distorted speech streams on neural activation during comprehension (Adank and Devlin, 2010; Eisner et al., 2010). Both studies monitored participants' neural activations while they perceptually adapted to distorted speech. Adank and Devlin presented participants with 64 sentences spoken by at a normal speed, before playing 64 sentences that had been artificially speeded up (time-compressed speech) to 45% of their original duration (Dupoux and Green, 1997). Participants performed a speeded sentence-verification task (i.e., deciding whether a sentence such as “Cars have four wheels” is true or false). When presented with the time-compressed sentences, performance on the sentence-verification task initially deteriorated sharply, but it quickly improved, signaling successful perceptual adaptation. Four cortical areas showed a pattern in the BOLD-activations in line with the behavioral results: one area in left ventral premotor cortex, two left lateralized areas in STG/STS, and one right-lateralized area in STG/STS. Eisner et al. studied the cortical changes in activity related to learning to perceptually adapt to noise-vocoded speech (Shannon et al., 1995). They included two types of distortion: one that was learnable and another was not Eisner et al. found that activity in left IFG correlated positively with individual variation in working memory scores, and that left IFG and left STS were sensitive to the extent to which the stimulus displayed learnable variation.

Nevertheless, a difference between the present study and Adank and Devlin's is that Adank and Devlin used an online learning design. They monitored neural responses as participants perceptually adapted. In the present study, we used an offline design: we did not monitor participants' neural responses during the training, to avoid problems with head motion during speaking. Consequently, it is possible that left IFG, STS, and SMA were even more active during the training phase for the imitation group. Adank and Devlin report that areas that showed an increase in their BOLD-response when participants were first confronted with the distortion (time-compressed sentences) in the speech signal also remained more active after participants had adapted. Thus, speech perception and speech production regions continued to be active when the listening conditions remained challenging.

However, others have suggested that other areas other than IFG, SMA, and STS are the focus of adaptation-related changes in speech perception. For instance, Rauschecker and Scott (2009) suggest hat the IPL provides an interface “where feed-forward signals from motor preparatory networks in the inferior frontal cortex and premotor cortex can be matched with feedback signals from sensory areas” (p. 722). Increased BOLD-activation was found—albeit at a lowered significance level—for the repeat group in the second run for the contrast [Repeat Run 2 > 1] > [Imitation Run 2 > 1]. However, no activation was found in IPL for the imitation group in the second run compared to the repeat group. It is possible that IPL was more active during the training phase for the imitation group and that the current design has not been able to record this activity as the scanner was turned off during training.

The question of which areas are active while participants are acquiring motor experience may be resolved in an online imitation design, for instance in an experiment in which participants are scanned while vocally imitating or repeating a novel speech stream. Such a design may allow for scrutiny of imitation-related activity in IPL, IFG, STS, and SMA.

### Interaction between group and run

The absolute pattern of results for the interaction effect observed in the behavioral data as well as in the neuroimaging data was not completely in line with our predictions. Namely, while we hypothesized an interaction in participants' performance between Run × Group, we expected that interaction to be driven by a significant increase in performance in Run 2 for participants who had imitated in contrast to participants who repeated in their own accent. Instead, the interaction in the current data set is driven by a relative decrease in performance for participants in the Repeat as compared to the Imitate Group. Thus, while the overall and relative pattern of results is in line with our predictions, the pattern within the second alone warrants further discussion.

The first explanation of these results is in terms of the simulation theories discussed in the Introduction. Simulation models presume that imitative motor involvement helps perceivers anticipate other people's actions better by generating forward models (Grush, 2004). These models serves to anticipate others' actions as if they were our own, something which is proposed to be beneficial for ambiguous or noisy signals or actions (Wilson and Knoblich, 2005; Pickering and Garrod, 2007). Simulation models predict that imitation improves perception, and several studies have recently confirmed this prediction for manual actions (Locatelli et al., 2012) and for speech (Adank et al., 2010). Simulation models generally presume that imitation is predominantly covert (and that any overt imitative action is actively suppressed, cf. Baldissera et al., 2001), but it is unclear how the act of overt motor imitation can support perception.

Moreover, recent behavioral research on perceptual learning in speech indicates that listeners adapt to accented speech by updating their internal phoneme representations for the speech sounds in question (e.g., Norris et al., 2003; Evans and Iverson, 2004). Information from speech articulators may be used to inform feed forward models used in the simulation process. Two recent studies support the possibility of such a supporting mechanism. D'Ausilio et al. (2009) demonstrated that repetitive Transcranial Magnetic Stimulation (rTMS) to lip and tongue motor cortex shifted perception of speech toward speech sounds involving these articulators, while Ito et al. (2009) demonstrated that perception of vowel sounds is affected by feedback from facial muscles while speaking these vowels with an altered pronunciation. Information from muscles can therefore, be used to inform perception. This information could be used by the forward models to improve prediction of utterances in the unfamiliar accent. In contrast, in the repeat group no updated information was available from motor neurons in articulation muscles. In turn, this could have resulted in the trend toward poorer comprehension after training in the current experiment.

However, an alternative explanation of the interaction between group and run and the trend toward lower intelligibility judgments in repeat group could be that participants in the repeat group performed a less engaging task. It seems plausible that having to replicate the way in which a sentence is being said is a task that requires more attention and effort than a task in which participants had to pronounce what was being said in their own accent. This possible increased lower engagement in the Repeat group may have had the following two consequences.

First, participants in the Repeat group could have gotten more fatigued and bored than those in the Imitation group. Consequently, participants in the Repeat group may not have been able (or willing) to pay as much attention in the first run. There exists anecdotal and experimental support for the notion that it is harder to maintain attention in unchallenging, monotonous tasks than for cognitively demanding but interesting tasks (see Robertson and O'connell, 2010, for an overview). Generally, behavioral performance deteriorates for longer sustained tasks, and this deterioration is faster for unchallenging tasks (such as repeating compered to imitating). A recent meta-analysis of neuroimaging studies on vigilant attention (i.e., the cognitive function that enables prolonged engagement in intellectually unchallenging, uninteresting activities) identified a network of—mostly right-lateralized—brain regions subserving vigilant attention, including dorsomedial, mid- and ventrolateral prefrontal cortex, anterior insula, parietal areas (intraparietal sulcus, temporoparietal junction), and finally subcortical structures (cerebellar vermis, thalamus, putamen, and midbrain) (Langner and Eickhoff, 2013). Note that our network of areas displaying the interaction effect ([Imitation Run 2 > 1] > [Repeat Run 2 > 1]) was exclusively left-lateralized and does not appear to overlap with the network identified by Langner and Eickhoff. Furthermore, inspecting of the reverse contrast ([Repeat Run 2 > 1] > [Imitation Run 2 > 1]) at a less stringent significance level indicated involvement of left IPL, whereas Langner and Eickhoff's analysis showed no clusters in the left parietal lobe.

Second, participants in the Repeat group performed a task that may have not allowed them to engage with the speaker of the accent as much as participants in the Imitation group. This decreased engagement could then have made them less willing to put as much effort into understanding the accented sentences in the second run as in the first run. This notion is speculative, but is supported by an experiment on the effect of imitating a target's (such as a speaker) behavior on the perception of the social characteristics of that target. In a within-subject design, Adank et al. (2013) asked a group of participants to listen to sentences spoken by two speakers of a regional accent (Glaswegian) of British-English. Next, they repeated the sentences spoken by one of the Glaswegian speakers in their own accent, and subsequently participants imitated sentences spoken by the second Glaswegian speaker. The order of repeating and imitating was counterbalanced across participants. After each repeating or imitation session, participants completed a questionnaire probing the speakers' perceived power, competence, and social attractiveness. Imitating had a positive effect on the perceived social attractiveness of the speaker compared to repeating. The authors explained the positive results of imitating by stating that the act of imitating another's accent makes the speaker part of participants' social in-group in a way that repeating does not. Since people are more positively biased toward people in their in-group than those outside (Brewer, 1979), such in-group favoritism then made the speaker seem more subjectively pleasant. However, it is unclear whether a comparable effect of imitation on perception of the speaker would also occur for the constructed accent used in our study. Hearing accented speech automatically invokes attitudes associated with speakers of the accent (Giles, 1970; Bishop et al., 2005; Coupland and Bishop, 2007) and it seems unlikely that constructed accents are associated with specific language attitudes as is the case for existing accents.

The issue of differences in engagement with the task across groups issue may be addressed in future studies by explicitly matching training tasks for “level of interest.” One way to achieve this would be to create two different constructed accents that are matched for intelligibility and have participants repeat sentences for one of the accents and imitate for the other accent in a within-subject design. Subsequently, their intelligibility of both accents could be tested to verify that imitation had a positive effect on the intelligibility of the imitated constructed accent. However, we chose not to take this route in the present study, as we aimed to replicate the design in Adank et al. (2010) as closely as possible using an fMRI design. Yet, future studies could, for instance, pre-test a variety of tasks on how much they are perceived to be equally interesting or engaging by participants or use multiple constructed accents.

A final explanation for the pattern in the behavioral results could be that the behavioral task used in the experiment, perhaps in combination with the presence of continuous scanner noise, did not accurately reflect the extent to which participants' performance changed over the two runs. Our earlier behavioral study described in Adank et al. (2010) used the standard SRT procedure (which is the same as described for the hearing test in the Materials and Methods section), and it seems plausible that this test provides a more fine-grained measure of intelligibility processing than the test used in the scanner. Adank et al.'s SRT procedure involved overt vocal responses, which was not optimal given our fMRI design. Future studies should thus, consider using a more fine-grained behavioral task, possibly in combination with a sparse sampling design (Hall et al., 1999).

### Conclusion

Previous studies (e.g., Davis and Johnsrude, 2003; Adank, 2012) have shown that processing of intelligible speech involves neural substrates associated with speech production and speech perception. This study adds to a growing body of literature showing that processing of intelligible speech involves sensorimotor processes. However, further research is required to establish in what manner overt imitation of speech contributes to these sensorimotor processes in challenging listening situations. Ideally, future studies involving imitation related-training should make a point of explicitly disentangling the possible contribution of factors such as participant (dis)engagement and listening environment. Only by addressing these issues can the contribution of imitative actions to speech perception be elucidated.

### Conflict of interest statement

The authors declare that the research was conducted in the absence of any commercial or financial relationships that could be construed as a potential conflict of interest.

## Appendix

**Table A1:** **Test sentences from the Speech Reception Threshold corpus (Plomp and Mimpen, 1979)**.

**Nr**.	**Standard Dutch**	**Accented Dutch**
1	De bal vloog over de schutting	De baal vlog offer de schuuttieng
2	Morgen wil ik maar één liter melk	Moorgen wiel iek mar èn litter meelk
3	Deze kerk moet gesloopt worden	Desse keerk mut geslopt woorden
4	De spoortrein was al gauw kapot	De sportreen waas aal goew kaappoot
5	De nieuwe fiets is gestolen	De niwwe fits ies gestollen
6	Zijn manier van werken ligt mij niet	Zeen mannir vaan weerken liegt mee nit
7	Het slot van de voordeur is kapot	Het sloot vaan de vordur ies kaappoot
8	Dat hotel heeft een slechte naam	Daat hotteel heft ‘n sleechte nam
9	De jongen werd stevig aangepakt	De joongen weerd steffig angepaakt
10	Het natte hout sist in het vuur	Het naatte hoet siest ien het vur
11	Zijn fantasie kent geen grenzen	Zeen faantassih keent gèn greenzen
12	De aardappels liggen in de schuur	De ardaappels liegen ien de schur
13	Alle prijzen waren verhoogd	Aalle preezen warren verhogt
14	Zijn leeftijd ligt boven de dertig	Zeen lèfteed liegt boffen de deertieg
15	Het dak moet nodig hersteld worden	Het daak mut noddieg heersteeld woorden
16	De kachel is nog steeds niet aan	De kaachel ies noog stèds nit an
17	Van de viool is een snaar kapot	Vaan de vij-jol ies ‘n snar kaappoot
18	De tuinman heeft het gras gemaaid	De tuunmaan heft het graas gemajt
19	De appels aan de boom zijn rijp	De aappels an de bom zeen reep
20	Voor het eerst was er nieuwe haring	Vor het erst waas eer niwwe harrieng
21	Het loket bleef lang gesloten	Het lokkeet blef laang geslotten
22	Er werd een diepe kuil gegraven	Eer weerd 'n dippe koel gegraffen
23	Zijn gezicht heeft een rode kleur	Zeen geziecht hèft 'n rodde klur
24	Het begon vroeg donker te worden	Het beggoon vrug doonker te woorden
25	Het gras was helemaal verdroogd	Het graas waas hèllemal verdrogt
26	Spoedig kwam er een einde aan	Spuddieg kwaam eer ‘n eende an
27	Ieder half uur komt hier een bus langs	Idder haalf ur koomt hir ‘n buus laangs
28	De bel van de voordeur is kapot	De beel vaan de vordur ies kaappoot
29	De wind waait vandaag uit het westen	De wiend wajt vaandag uut het weesten
30	De slang bewoog zich door het gras	De slaang bewog ziech dor het graas
31	De kamer rook naar sigaren	De kammer rok nar siggarren
32	De appel had een zure smaak	De aappel haad 'n zurre smak
33	De trein kwam met een schok tot stilstand	De treen kwaam meet ‘n schook toot stielstaand
34	De koeien werden juist gemolken	De kujjen weerden juust gemoolken
35	Het duurt niet langer dan een minuut	Het durt nit laanger daan ‘n minnut
36	De grijze lucht voorspelt regen	De greeze luucht vorspeelt règgen
37	Hij kon de hamer nergens vinden	Hee koon de hammer neergens vienden
38	Deze berg is nog niet beklommen	Desse beerg ies noog nit bekloommen
39	De bel van mijn fiets is kapot	De beel vaan meen fits ies kaappoot
40	De auto heeft een lekke band	De oetoh hèft ‘n leekke baand
41	Het moeilijke werk bleef liggen	Het muj-leekke weerk blef lieggen
42	Het vliegtuig vertrekt over een uur	Het vligtuug vertreekt offer ‘n ur
43	De jongens vechten de hele dag	De joongens veechten de hèlle daag
44	De schoenen moeten verzoold worden	De schunnen mutten verzold woorden
45	In de krant staat vandaag niet veel nieuws	Ien de kraant stat vaandag nit vèl niws
46	Door de neus ademen is beter	Dor de nus addemmen ies better
47	Het kind was niet in staat te spreken	Het kiend waas nit ien stat te sprekken
48	De witte zwaan dook onder water	De wiette zwan dok oonder watter
49	Hij nam het pak onder zijn arm	Hee naam het paak oonder zeen aarm
50	Gelukkig sloeg de motor niet af	Geluukkieg slug de mottor nit aaf
51	De leraar gaf hem een laag cijfer	De lèrrar gaaf heem ‘n lag seeffer
52	Het huis brandde tot de grond toe af	Het huus braande toot de groond tuh aaf
53	De foto is mooi ingelijst	De fotto ies moi iengeleest
54	Mijn broer gaat elke dag fietsen	Meen brur gat eelke daag fitsen
55	Een kopje koffie zal goed smaken	Een koopje kooffih zaal gud smakken
56	De schrijver van dit boek is dood	De schreeffer vaan diet buk ies dot
57	Zij heeft haar proefwerk slecht gemaakt	Zee heft har prufweerk sleecht gemakt
58	De sigaar ligt in de asbak	De siggar liegt ien de aasbaak
59	De appelboom stond in volle bloei	De aappelbom stoond ien voolle bluj
60	Er wordt in dit land geen rijst verbouwd	Eer woordt ien diet laand gèn reest verbuwd
61	Hij kan er nu eenmaal niets aan doen	Hee kaan eer nuh ènmal nits an dun
62	De kleren waren niet gewassen	De klerren warren nit gewaassen
63	Het gedicht werd voorgelezen	Het gediecht weerd vorgelèssen
64	Haar gezicht was zwart van het vuil	Har geziecht waas zwaart vaan het vuul
65	De letters stonden op hun kop	De leetters stoonden oop huun koop
66	De groene appels waren erg zuur	De grunne aappels warren eerg zur
67	In het gebouw waren vier liften	Ien het geboew warren vir lieften
68	Lopen is gezonder dan fietsen	Loppen ies gezoonder daan fitsen
69	Het lawaai maakte hem wakker	Het lawwai makte heem waakker
70	Mijn buurman heeft een auto gekocht	Meen burmaan heft ‘n oetoh gekoocht
71	Als het flink vriest kunnen we schaatsen	Aals het flienk frist kuunnen we schatsen
72	De kast was een meter verschoven	De kaast waas 'n metter verschoffen
73	Oude meubels zijn zeer in trek	Oede mubbels zeen zèr ien treek
74	De portier ging met vakantie	De poortir gieng meet vaakkaantih
75	De lantaarn gaf niet veel licht meer	De laantarn gaaf nit vèl liecht mer
76	Door zijn snelheid vloog hij uit de bocht	Door zeen sneelheed vlog hee uut de boocht
77	Het is hier nog steeds veel te koud	Het ies hir noog steds vèl te koed
78	De oude man was kaal geworden	De oede maan waas kal gewoorden
79	De bomen waren helemaal kaal	De bommen warren hèllemal llemal kal
80	Rijden onder invloed is strafbaar	Reedden oonder ienvlud ies straafbar
81	Onze bank geeft vijf procent rente	Oonze baank geft veef prosseent reente
82	Het verslag in de krant is kort	Het verslaag ien de kraant ies koort
83	In de vijver zwemmen veel vissen	Ien de veeffer zweemmen vel viessen
84	Honden mogen niet in het gebouw	Hoonden moggen nit ien het geboew
85	Een flinke borrel zal mij goed doen	Een flienke boorrel zaal mee gud dun
86	Gisteren waaide het nog harder	Giesteren wajde het noog haarder
87	Het meisje stond lang te wachten	Het meesje stoond laang te waachten
88	De volgende dag kwam hij ook niet	De voolgende daag kwaam hee ok nit
89	Het geschreeuw is duidelijk hoorbaar	Het geschrew ies duudeleek horbar
90	Eindelijk kwam de trein op gang	Eendeleek kwaam de treen oop gaang
91	De grote stad trok hem wel aan	De grotte staad trook heem weel an
92	De bus is vandaag niet op tijd	De buus ies vaandag nit oop teed
93	Onze dochter speelt goed blokfluit	Oonze doochter spèlt gud blookfluut
94	Ook in de zomer is het hier koel	Ok ien de zommer ies het hir kul
95	Zij moesten vier uur hard werken	Zee musten vir ur haard weerken
96	Niemand kan de Fransman verstaan	Nimmaand kaan de Fraansmaan verstan

Only vowels bearing primary or secondary lexical stress were included in the conversion of the orthography.

**Table A2:** **Training sentences**.

**Nr**.	**Standard Dutch**	**Accented Dutch**
1	Architecten hebben een beroep	Aarchitteecten heeben een berup
2	Asperges zijn groenten	Aaspeerges zeen grunten
3	Aardappels worden geschild	Ardaappels woorden geschield
4	Bananen zijn fruit	Bannannen zen fruut
5	Bijen vliegen rond op zoek naar voedsel	Beej-jen vliggen roond oop zuk nar vudsel
6	Bevers bouwen dammen in de rivier	Beffers buwwen damen ien de rivvir
7	Beren hebben vier poten	Berren heeben vir potten
8	Bisschoppen dragen kleren	Biesschopen draggen klerren
9	Biefstukken worden verkocht door slagers	Bifstuukken woorden verkoocht dor slaggers
10	Blikopeners kunnen in winkels gekocht worden	Bliekoppeners kuunen ien wienkels gekoocht woorden
11	Bromfietsen rijden op de weg	Broomfitsen reeden oop de weeg
12	Chirurgen moeten lang studeren	Chieruurgen mutten laang studderren
13	Druiven zijn eetbaar	Druuven zeen etbar
14	Ezels dragen zware vrachten	Essels draggen zwarre vraachten
15	Ezels kunnen koppig zijn	Essels kuunen kooppieg zeen
16	Forellen hebben schubben	Forrelen heeben schuubben
17	Ganzen kunnen ver vliegen	Gaanzen kuunen veer vliggen
18	Haaien hebben sterke tanden	Hajjen heeben steerke taanden
19	Heggenscharen worden in de tuin gebruikt	Heegenscharren woorden ien de toen gebroekt
20	Kapiteins voeren het bevel op schepen	Kaapitteins vurren het bevveel oop scheppen
21	Kasten zijn van hout	Kaasten zeen vaan hoet
22	Kroketten zijn gefrituurd	Krokkeetten zeen gefritturd
23	Lammetjes komen van schapen	Lametjes kommen vaan schappen
24	Lepels worden gebruikt voor het eten van soep	Leppels woorden gebroekt vor het etten vaan sup
25	Leeuwen hebben manen	Lewwen heeben mannen
26	Luipaarden hebben een vacht	Luuparden heeben ‘n vaacht
27	Makrelen ademen door kieuwen	Maakrellen addeemen dor kiwwen
28	Messen worden gebruikt als keukengerei	Meessen woorden gebroekt aals kukkengeree
29	Monniken wonen in een klooster	Moonnieken wonnen ien ‘n kloster
30	Nachtegalen hebben veren	Naachtegallen heeben verren
31	Ooms zijn deel van de familie	Oms zeen del vaan de fammillih
32	Otters kunnen goed zwemmen	Ooters kuunen gut zweemen
33	Pinguïns eten veel vis	Piengiens etten vel vies
34	Presidenten werken in de politiek	Pressideenten weerken ien de pollittik
35	Ratelslangen kruipen op hun buik	Rattelslaangen kroepen oop huun boek
36	Roodborstjes hebben een snavel	Rodboorstjes heeben een snaffel
37	Schuurtjes worden gebruikt voor opslag	Schurtjes woorden gebroekt vor oopslaag
38	Slagers hebben winkels	Slaggers heeben wienkels
39	Sloffen worden gemaakt in een fabriek	Sloofen woorden gemakt ien ‘n fabbrik
40	Tantes zijn altijd vrouwen	Taantes zeen aalteed vroewen
41	Tijgers hebben een staart	Teegers heeben ‘n start
42	Tomaten groeien aan planten	Tommatten grujjen an plaanten
43	Vaders zijn ouders	Vadders zeen oeders
44	Vlinders hebben voelsprieten	Vlienders heeben vulspritten
45	Wandelschoenen zijn gefabriceerde goederen	Waandelschunnen zeen gefaabrisserde gudderen
46	Wijflessen hebben een kurk	Weenfleessen heeben ‘n kuurk
47	Wetenschappers moeten lang studeren	Wettenschaapers mutten laang studderren
48	Wortels groeien in een moestuin	Woortels grujjen ien ‘n mustuun

Only vowels bearing primary or secondary lexical stress were included in the conversion of the orthography.

**Table A3:** **Hearing test sentences, spoken in Standard Dutch**.

**Nr**.	**Standard Dutch**	**Key words**
1	Het regent al de hele dag	regent al hele dag
2	De schaatsen zijn in het vet gezet	schaatsen zijn vet gezet
3	In juni zijn de dagen het langst	juni zijn dagen langst
4	De bakkers bezorgen vandaag niet	bakkers bezorgen vandaag niet
5	Hij rookte zijn sigaret op	rookte zijn sigaret op
6	Het was heel stil in de duinen	was heel stil duinen
7	Door zijn haast maakte hij veel fouten	Door haast veel fouten
8	De kat likt het schoteltje leeg	kat likt schoteltje leeg
9	De hond blafte de hele nacht	hond blafte hele nacht
10	Het tempo lag voor hem veel te hoog	tempo hem veel hoog
11	Lawaai maakt je op den duur doof	Lawaai maakt duur doof
12	Moeizaam klom de man naar boven	Moeizaam klom man boven
13	Hij probeerde het nog een keer	Hij probeerde nog keer
14	Toch lijkt me dat een goed voorstel	Toch lijkt goed voorstel
15	Dat was voor hem een bittere pil	Dat hem bittere pil
16	De nieuwe zaak was pas geopend	nieuwe zaak pas geopend
17	De rivier trad buiten haar oevers	rivier trad buiten oevers
18	De biefstuk was vandaag erg mals	biefstuk vandaag erg mals
19	Op het gras mag men niet lopen	gras mag niet lopen
20	De trein vertrekt over twee uur	trein vertrekt twee uur
21	De kat van de buren is weg	kat van buren weg
22	De wagen reed snel de berg af	wagen reed snel berg
23	Iedereen genoot van het uitzicht	Iedereen genoot van uitzicht
24	Steile trappen zijn gevaarlijk	Steile trappen zijn gevaarlijk
25	De zon gaat in het westen onder	zon gaat westen onder
26	De zak zat vol oude rommel	zak vol oude rommel
27	Zij werd misselijk van het rijden	Zij werd misselijk rijden
28	Het licht in de gang brandt nog steeds	licht gang brandt steeds
29	In de kerk werd mooi orgel gespeeld	kerk mooi orgel gespeeld
30	De jongens gingen er gauw vandoor	jongens gingen gauw vandoor
